# 

**Published:** 1909-03-15

**Authors:** 


CONTENTS

Event and Comment - - - - - - - - 109

Latest Developments in the Cast Gold Process,
*By Albert L. LeGro, D.D.S.* 114

Decayed Teeth and Appendicitis; Points the Pupils of Our Public Schools Should Know Regarding Them,
*By W. Guy Smith, D.D.S.* 134

To Prevent Strangling During Extraction,
*By F. H. Skinner, D.D.S.* 138

Treatment of Interstitial Gingivitis,
*By Eugene S. Talbot, M.S., D.D.S., M.D., L.L.D.* 138

Obituary - - - - - - - - - - 142

Indents - - - - - - - - - - - - 144

Announcements - - - - - - - - - - 155

